# Rank for Our Army Veterinarians

**Published:** 1896-01

**Authors:** 


### RANK FOR OUR ARMY VETERINARIANS.

In this number we print a copy of the bill introduced in Congress for the recognition of the veterinarian. In addition, the names of the committee representing the United States Veterinary Medical Association and lists of the Committees on Military Affairs in the Senate and House. The time has gone by for discussion of the merits of this question; its failure in the past, no matter from what cause or causes, remains to-day a blot on the escutcheon of our profession, an open point of attack for just criticism of our government in military affairs, when compared with that of every intelligent, progressive nation of the world; a misfortune to our people, in that they are denied the widespread benefits to be derived by every step higher they raise one of the most important sciences on earth. A delusive form of economy, by which our government is denied the highest and most intelligent aid in this service, where so much might be saved in a monetary sense were rank and with it a wider latitude for the exercise of a greater degree of intelligent selection and purchase conceded, to those only who are fitted to render well these services.

Since the last effort to obtain this recognition failed, State after State has enacted laws looking to the greater safeguards always to be obtained by a higher degree of intelligence upon the part of her servants and boards for the selection of better-equipped men have been created. Boards for the keener exercise of vigilance in saving our people from the dangers of an impure food-supply, local and State, engrafted upon many sections of our country, all of which betoken the spirit of the age —advancement.

The committee who have taken this bill in charge, while created by the powers of the National Association, are well known and thoroughly representative professional men in every way, and stand ready, willing, determined, to fully represent the entire profession in zealous work to accomplish this much-to-be-desired end. Already in harness, they have made the first stroke a telling one, and won for the bill the friendly influences of those who are charged with the keeping of these affairs for our people. But this is not enough; they must have back of them the entire profession of the country, and we call upon every member to aid them by using their influence with those who represent them in either branch of Congress. Letters, personal interviews, are all to be used and put into force at once, not next week, next month, but to-day, and in such language that these servants of the people will understand that you are alive to the needs of your adopted calling, and are not afraid to advocate them when you know that the greater benefits are to be derived by those who receive these services. The need of the hour is work, and the call has gone forth; everyone has his influence; let it be wielded.

